# Fisetin inhibits proliferation of pancreatic adenocarcinoma by inducing DNA damage via RFXAP/KDM4A-dependent histone H3K36 demethylation

**DOI:** 10.1038/s41419-020-03019-2

**Published:** 2020-10-22

**Authors:** Guoping Ding, Xiaodong Xu, Dan Li, Yuhao Chen, Weimin Wang, Dongnan Ping, Shengnan Jia, Liping Cao

**Affiliations:** 1 Department of General Surgery, Sir Run Run Shaw Hospital, School of Medicine, Zhejiang University, Hangzhou, 310000 China; 2 Department of General Surgery, School of Medicine, Affiliated Hospital of Hangzhou Normal University, Hangzhou, 310000 China; 3 Emergency Department, Sir Run Run Shaw Hospital, School of Medicine, Zhejiang University, Hangzhou, 310000 China; 4 Department of General Surgery, Huzhou Hospital, Zhejiang University School of Medicine, Huzhou, 313003 Zhejiang China; 5 Innovation Center for Minimally Invasive Technique and Device, Zhejiang University, Hangzhou, 310000 Zhejiang China

**Keywords:** Cancer epigenetics, Drug development, Pancreatic cancer

## Abstract

Pancreatic adenocarcinoma (PDAC) is an extremely malignant tumor that is associated with low survival rates. Fisetin is a natural flavonoid that shows diverse antitumor effects, including DNA damage, in various cancers. Increasing studies have demonstrated that epigenetic modifications play critical roles in DNA-damage response. However, the epigenetic regulation mechanism of fisetin in cancers is hardly studied. RFXAP is a critical transcription factor for MHC II molecules, however, its transcriptional role in PDAC is poorly understood. The anti-PDAC effect of fisetin was measured by CCK-8, flow cytometry, xenograft tumor nude mice model. DNA-damage levels were examined by immunofluorescence. Bioinformatics analysis was used to examine the expression of RFXAP and other genes involved in DNA-damage response. ChIP sequencing was used to explore the transcriptional role of RFXAP. The expression of target gene *KDM4A* was measured by qRT-PCR and western blots. *KDM4A* promoter activity was analyzed using dual-luciferase reporter assay. RFXAP overexpressing or silencing of PDAC cells was used to explore the effect of RFXAP in DNA damage induced by fisetin. We found that fisetin inhibited cell proliferation and induced DNA damage and S-phase arrest in PDAC. Expression of *RFXAP* and other DNA-damage response genes were upregulated by fisetin. We revealed that *RFXAP* expression was relatively low in PDAC and correlated with tumor stage and poor prognosis. Then we explored the transcriptional role of RFXAP and found that RFXAP targeted *KDM4A*, a special demethylase specific for tri- and dimethylated histone H3K36. We found that overexpression of RFXAP upregulated *KDM4A* and attenuated methylation of H3K36, thereby impairing DNA repair and enhancing the DNA damage induced by fisetin, while *RFXAP* silencing showed the opposite effect. We also found the function of fisetin in enhancing the effect of chemotherapy on pancreatic cancer cells. Our findings revealed that fisetin induced DNA damage via RFXAP/KDM4A-dependent histone H3K36 demethylation, thus causing inhibition of proliferation in PDAC.

## Introduction

Pancreatic adenocarcinoma (PDAC) is an extremely malignant tumor characterized by low rates of early diagnosis and radical resection^1^. Patients with PDAC frequently develop resistance to chemotherapy and radiotherapy, and the prognosis of these patients is poor, with 5-year survival rates of 3–6%^2^. It is the fourth leading cause of cancer-related death in the United States and the fifth in China^3,4^.

Because of uncontrolled proliferation, cancer cells are more likely to incur DNA damage including double-strand breaks (DSBs) and single-strand breaks^5^. Excessive DSBs beyond the capacity for cellular repair, induced by radiation or chemotherapeutic drugs such as cisplatin and etoposide, cause death of replicating cells; however, cancer cells with elevated DNA repair mechanisms may have acquired and intrinsic drug resistance, and thus poor chemotherapy outcomes^6,7^. Recent studies have demonstrated that inhibition of DNA repair pathways in cancer cells can significantly enhance the effects of chemotherapy and radiotherapy^8,9^.

H3K36 methylation plays a conserved role in regulating DNA repair in cancer. Recent studies have demonstrated that SETD2-dependent H3K36me3 is critical for homologous recombination (HR) repair^10,11^. Interestingly, the trimethylation of H3K36 is not induced by DNA damage but is pre-existing^12^. KDM4A is a demethylase specific for tri- and dimethylated histone H3K36 and H3K9, which causes demethylation of histone H3K36 and thereby impairs HR^10^.

Regulatory factor X-associated protein (RFXAP) is a critical transcription factor for MHC II molecules^13,14^. We previously found that miR-212-3p transfers from pancreatic cancer cells to dendritic cells via exosomes, inhibiting expression of RFXAP and major histocompatibility complex (MHC) class II molecules and leading to immune tolerance of dendritic cells^15^ miR-212-3p is upregulated in PDAC and is correlated with TNM stage and poor prognosis^16–18^. Thus, we hypothesized that miR-212-3p-induced RFXAP deficiency may occur in PDAC. Deficiency of RFXAP can lead to inactivation of CD4+ T lymphocytes, which is a rare and severe immunodeficiency disease termed bare lymphocyte syndrome^19^. However, the role of RFXAP deficiency in malignant tumors has not been documented.

Fisetin, as a natural flavonoid, is mainly present in various vegetables and fruits such as apples, cucumbers, onions, and strawberries^20^. Fisetin is known to possess diverse pharmacological activities, such as antioxidant^21^, anti-inflammatory^22^, and antitumor effects in various cell types^23–25^. From these reports, it appears that the antitumor mechanism of fisetin may be cancer-cell-specific. Recent studies reported that fisetin can induce DNA damage and apoptosis in gastric and hepatic cancer and enhance the DNA-damage-induced toxicity of etoposide^25–27^. However, there have been no studies that focused on the DNA damage induced by fisetin in PDAC.

In this study, we found that the expression of RFXAP was relatively low in pancreatic cancer, and its downregulation was correlated with tumor stage and poor prognosis. RFXAP was shown to bind to the promoter region of the *KDM4A* gene and induce its expression, affecting DNA repair. We have demonstrated that fisetin can induce DNA-damage cytotoxicity in PDAC through regulation of RFXAP/KDM4A-dependent demethylation of H3K36me3, thereby impairing DNA HR repair. The present results suggest that fisetin-induced RFXAP and KDM4A play an important role in antitumor processes by causing DNA damage and DNA repair dysfunction.

## Materials and methods

### Reagents and antibodies

Primary antibodies used in this study were as follows: polyclonal anti-ɑ-tubulin (Sigma, catalog no.T6199), anti-RFXAP (Abcam, ab9258; ab87251), anti-KDM4A (Abcam, ab191433), anti-H3 (Abcam, ab201456), anti-H3K36me3 (Abcam, ab9050), anti-γ-H2AX(Abcam, ab26350; Cell Signaling Technology, catalog no. #9718S).

### Cell culture

The human pancreatic cancer-cell lines BxPC-3, MiaPACA-2, PANC-1, and HPC-Y5 and the normal pancreatic ductal epithelial cell line HPDE6-C7 were obtained from the Chinese Academy of Sciences (Shanghai, China). BxPC-3 cells were cultured in RPMI 1640 medium (Hyclone) containing 10% fetal bovine serum (FBS, Gibco, New York, USA). PANC-1, HPC-Y5, MiaPACA-2, and HPDE6-C7 cells were cultured in DMEM supplemented with 10% FBS.

### Patients and tissue specimens

Forty-six PDAC patients who underwent surgery between 2012 and 2016 were selected from Sir Run Run Shaw Hospital, affiliated with the Zhejiang University of Medicine, China. All tissue specimens were diagnosed by pathological examination after surgery. The mean age of the patients was 59.0 years (range, 35–82). The male to female ratio was 22:24. The research protocol was reviewed and approved by the Research Ethics Committee of Sir Run Run Shaw Hospital, School of Medicine, Zhejiang University. All participants or their guardians gave written consent for the use of tissue samples and medical information to be used for scientific research.

### Clinicopathological parameters

Clinicopathological factors including age, gender, tumor size, differentiation, histological stage, lymph node metastasis, and distant metastasis were retrospectively analyzed. Survival data were reviewed in all cases. The histological stage of PDAC patients was assessed on the basis of the newest TNM staging system described by the American Joint Committee on Cancer (AJCC) in 2017.

### Immunohistochemistry

Immunohistochemical staining was performed using the standard streptavidin-biotin-peroxidase complex method. Briefly, paraffin-embedded tissue sections were dewaxed and rehydrated through an alcohol series, and endogenous peroxidase activities were blocked. Slides were incubated overnight at 4 °C with monoclonal anti-RFXAP (Abcam, ab87251). Samples incubated with PBS instead of primary antibody were used as the negative control. Proteins were detected using secondary antibodies and counterstained in Gill’s hematoxylin, then dehydrated in an ascending methanol series prior to clearing in xylene and mounting under a coverslip. The sections were observed under an Olympus CX31 microscope (Olympus, Tokyo, Japan).

Cells with the cytoplasm stained yellow to brown were scored as RFXAP immunopositive. RFXAP expression was classified semiquantitatively according to the following criteria: −, <1% of neoplastic cells discretely expressing RFXAP; +, ≥1 of morphologically unequivocal neoplastic cells discretely expressing RFXAP.

### Transfection and siRNA

For transduction, cells were transfected with the appropriate target plasmid and packaging constructs overnight, and the viral supernatant was collected 48 h later. PANC-1 and BxPC-3 cells were transduced in the presence of 5-μg/ml polybrene and selected for 3–7 days depending on the level of EGFP expression. *RFXAP* siRNAs (Ribobio, China) were used to knock down *RFXAP* gene expression. The *RFXAP* and control siRNA (100 pM) were transfected into PANC-1 cells using siRNA transfection reagent (Santa Cruz Biotechnology) 24 h before fisetin treatment. The cells were collected after fisetin treatment for 48 h. The Ubi-MCS-Luc-IRES-Puromycin vector was used to construct PANC-1-luciferase cells. Cells were transfected using Lipofectamine 3000 (Invitrogen, USA) and selected for puromycin resistance. *KDM4A*-knockout PANC-1 cells were constructed using the CRISPR/Cas9 gene-editing system. The target sequences of gRNAs were designed using the online tool (https://www.benchling.com/crispr/), which listed as follows: KDM4A-cas9 (5′-AAGGTCATTATCCTAGCACT-3′). Plasmid Mini-PREPS Kit (Sangon Biotech, catlogB110091-0100) was used for plasmid extraction. CRISPR plasmids were transfected using Lipofectamine 3000 (Invitrogen, USA) and selected for puromycin resistance.

### Dual-luciferase reporter assay

PANC-1 and BxPC-3 cells pre-transfected with pcDNA3.1(−) (Invitrogen, USA) or pcDNA-RFXAP (RFXAP overexpression) vectors were co-transfected with the pGL4.23-KDM4A promoter-luc reporter vector. pRL-TK vector (Promega, Madison, WI, USA) was used as an internal control. Cell lysates were harvested for the dual-luciferase assay, which was performed according to the manufacturer’s instructions (Promega, Madison, WI, USA).

### Animals

Nude mice were obtained from SLAC Laboratory and bred in the animal center of Zhejiang University under specific pathogen-free conditions. Orthotopic PDAC model was established with human pancreatic cancer PANC-1-luciferase cells. Tumor growth and the condition of mice were monitored every other day. Animal experiments were approved and performed in accordance with the institutional guidelines for animal care of the animal ethics committee of Zhejiang University. For the detection of bioluminescence, mice were injected intraperitoneally with 200 μL (15 mg/mL) of D-luciferin. Tumors were imaged after 15 min of biodistribution, with an IVIS Lumina Series III (Perkin Elmer, USA).

### Gene co-expression analysis

RNA-Seq data of 182 pancreatic cancer samples were collected from TCGA (http://cancergenome.nih.gov). The RNA-Seq data were transformed into log2 scale. Based on the correlation analyses, genes coexpressed with RFXAP were identified according to a Pearson’s correlation coefficient >0.5.

### ChIP-sequencing data analysis

FastQC was used as a stand-alone interactive application for the immediate analysis of FastQ files to determine whether the ChIP-seq data had any problems before the mapping analysis. The sequenced DNA tags (49 bp) were all sequence reads generated from Illumina HiSeq 2500 and were aligned against the human reference sequence (hg19, June 2011) using the Bowtie2 tool, allowing one mismatch in the default seed length of 49 bases. The sequence reads mapped to the genome were subjected to peak calling to detect regions with significant enrichment of ChIP signals with respect to the background by using MACS2. Motifs were searched both in the full list of peaks and in subsets of peaks divided by enrichment, and location, and sequences 2-kb upstream of the transcription start site were used as background. The DAVID software was used for Gene Ontology and Pathway analyses.

### Apoptosis and cell-cycle analysis

The levels of cell apoptosis were tested using the BD FITC Annexin-V Apoptosis Detection Kit I (556547). Cells were analyzed by flow cytometry after 1 h. Cell cycle was detected by the Cell-Cycle Staining Kit (MultiSciences, China, 70-CCS012).

### Immunofluorescence via confocal microscopy

PDAC cells were fixed with 4% paraformaldehyde at 4 °C overnight and then washed with PBS in 5 min for three times. Fixed cells were blocked in antibody dilution buffer (PBS containing 0.25% Triton X-100 and 1% bovine serum albumin), and all subsequent staining was performed in the same buffer. The cells were labeled with primary antibody for 2 h and subsequently with Alexa Fluor 488-conjugated donkey anti-rabbit secondary antibody (Invitrogen Life Technologies) for 1.5 h. The nucleus was stained by incubation with DAPI for 15 min at room temperature. Cells were scanned using a ZEISS LSM 880 confocal microscope (Zeiss, Jena, TH, GER).

### Statistical analysis

Data were analyzed using SPSS version 20.0. The Kaplan–Meier method was used to analyze patient survival, and the differences in survival were evaluated using the log-rank test. Multivariate analysis was performed using the Cox proportional hazards model to confirm factors independently associated with survival. The paired-samples *t*-test was used to compare the intensity ratio between HCC tissues and matching noncancerous liver tissues. All *P* values were based on two‑sided statistical analyses, and *P* < 0.05 was considered statistically significant.

## Results

### Fisetin inhibited the viability of human pancreatic cancer cells in vitro and in vivo

**Fig. 1:**
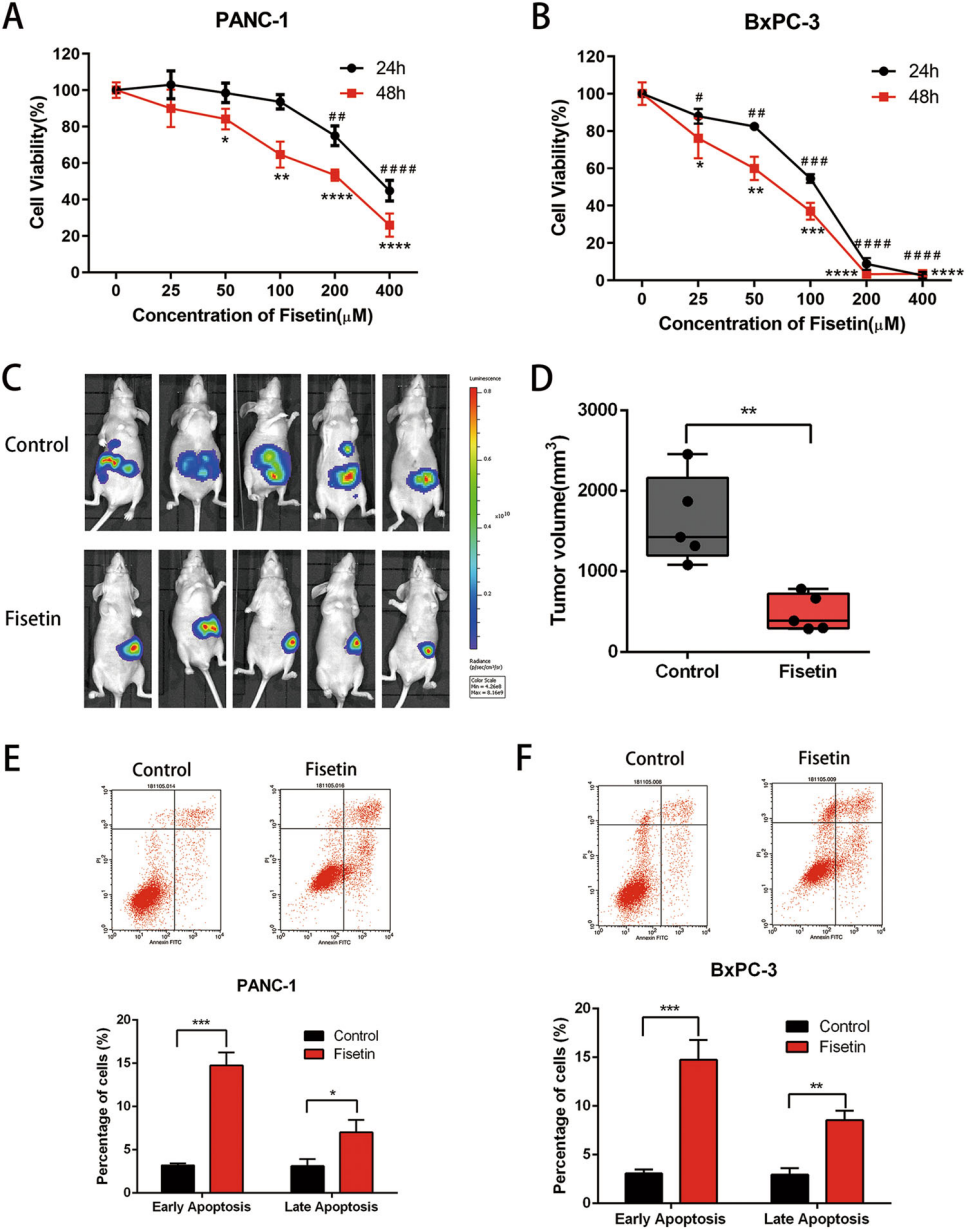
Effect of fisetin on viability of PDAC in vitro and in vivo. **a, b** CCK-8 asssy of PANC-1 and BxPC-3 cells. Cells were treated with fisetin (0–400 μM) for 24, 48 h, respectively. The absorbance was measured at 450 nm. Data are presented as mean ± SD (*n* = 3); * or #*P* < 0.05, ** or ##*P* < 0.01, *** or ###*P* < 0.001, **** or ####*P* < 0.0001. **c** Orthotopic PDAC model was established with human pancreatic cancer PANC-1-luciferase cells. Control group mice were treated with DMSO via intraperitoneal injection, and treatment group mice were treated with Fisetin (160-mg/kg body weight i.p.) every other day. After 30 days treatment, mice imaged for bioluminescence. Data were expressed as average radiance (photons/s/cm^2^/sr). **d** The differences in tumor volume between control and fisetin treatment groups at 28 days after treatment. Results are mean ± SD (*n* = 5), **P < 0.01. **e, f** Apoptosis of cells with treatment. PANC-1 and BxPC-3 cells were treated with fisetin (100, 50 μM) for 48 h, respectively, followed by Annexin-V/PI staining and flow cytometry analysis. Data are presented as mean ± SD (*n* = 3); *P < 0.05,**P < 0.01, ***P < 0.001.

We measured the effect of fisetin on the proliferation and growth of four pancreatic cancer-cell lines (PANC-1, BxPC-3, MiaPACA-2, HPC-Y5) using a Cell Counting Kit-8 assay. Cancer cells were cultured with increasing concentrations of fisetin (0, 25, 50, 100, 200, and 400 µM), and the results showed that fisetin reduced pancreatic cancer-cell viability in a dose-dependent manner (Fig. 1a, b and Supplementary Fig. S1A, B). Interestingly, we found that low concentrations of fisetin (25, 50 µM) did not inhibit the viability of HPC-Y5 and PANC-1 cells efficiently, while BxPC-3 and MiaPACA-2 were sensitive to low concentration of fisetin. Thus, we hypothesized that the differences in sensitivity to fisetin may be cell-specific.

Although studies on the antitumor effect of fisetin have increased over recent years, there have been few studies about its in vivo antitumor activity, especially in PDAC. To determine the in vivo efficacy of fisetin, we selected PANC-1 cells with low sensitivity to fisetin in vitro and used a nude mouse xenograft model of luciferase-expressing PANC-1 tumor cells, which emit bioluminescence after intraperitoneal luciferin injection. This allowed the noninvasive detection of tumor size and position in living mice. Notably, fisetin significantly reduced tumor size in mice (Fig. 1c). The tumor volumes 30 d after treatment were 1631.0 ± 243.3 mm^3^ and 483.7 ± 100.7 mm^3^ (mean ± standard deviation) in the control and fisetin treatment groups, respectively (**P < 0.01; Fig. 1d). Although compared with BxPC-3 and MiaPACA-2, PANC-1 cells were not very sensitive to fisetin in vitro, the antitumor effects of fisetin were still significant. Overall, these results demonstrated that fisetin can significantly inhibit PDAC growth and invasion in vivo.

To investigate whether fisetin-induced PDAC cell death occurred via the apoptosis, we selected PANC-1 and BxPC-3 cells to assess phosphatidylserine translocation using Annexin-V and propidium iodide double-staining. Apoptosis analysis by flow cytometry showed a increased number of apoptotic cells in the fisetin treatment group (Fig. 1e, f). The proportion of cells of early apoptotic cells (Annexin-V positive) were 14.98% ± 0.67% (***P < 0.001) and 13.69% ± 0.89% (***P < 0.001) after 48 h of treatment with 100-µM fisetin for PANC-1 cells and 50-µM fisetin for BxPC-3 cells, respectively. These results demonstrated the ability of fisetin to induce apoptosis in PDAC cells.

### Fisetin induced S phase and DNA damage in pancreatic cancer cells

**Fig. 2:**
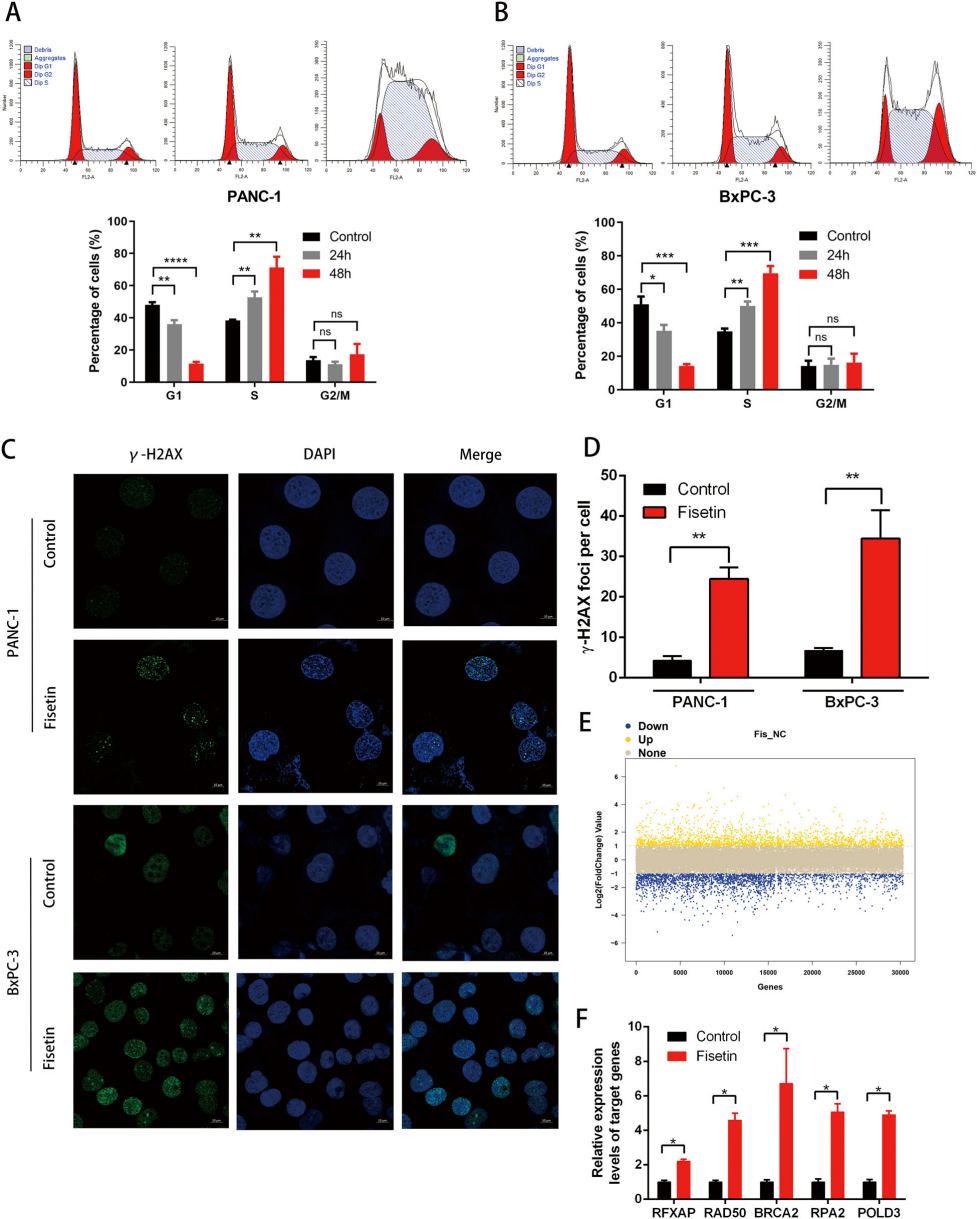
Fisetin induces S-phase arrest and DNA damage of PDAC. **a, b** Cell cycle of cells with treatment PANC-1 and BxPC-3 cells were treated with fisetin (100, 50 μM) for 48 h, respectively, followed by PI staining and flow cytometry analysis. Data are presented as mean ± SD (*n* = 3); *P < 0.05, **P < 0.01, ***P < 0.001, ****P < 0.0001, #ns no significance. **c** Analyses of DNA damage by observation of the immunofluorescence of γ-H2AX (green) in PDAC cells. PANC-1 and BxPC-3 cells were treated with fisetin (100, 50 μM) for 48 h, respectively. The nucleus were counterstained with DAPI (blue). Scale bars 10 μm. **d** Comparative changes in the γ-H2AX spots number in control and treatment groups. The spots were counted by manual scoring. Results are mean ± SD, **P < 0.01. **e** RNA-sequencing analysis identified 2672 differentially regulated genes (*P* < 0.05, *q* < 0.05, fold-change ≥ 2), of which 1136 and 1536 were up- and downregulated, respectively. #Fis fisetin treatment, NC negative control. Yellow and blue indicate high and low expression, respectively. **f** qRT-PCR detection of targeted genes in the RNA sequencing of PANC-1 cells. The results are presented as the mean ± SD (*n* = 3), *P < 0.05.

We found that fisetin also induced S-phase arrest in both PANC-1 and BxPC-3 cell lines. The results showed that time-dependent downregulation of G1-phase and upregulation of S-phase cells in both PANC-1 and BxPC-3 cells with treatment (Fig. 2a, b).

It is well known that many chemotherapeutic drugs such as gemcitabine and etoposide cause S-phase arrest and DNA damage, thereby inducing apoptosis. Thus, we hypothesized that fisetin might induce DNA damage in PDAC. To examine DNA-damage levels, we used immunofluorescence to detect gamma-H2AX foci, which is a DNA-damage marker (Fig. 2c). The results showed that gamma-H2AX foci levels were significantly increased in PDAC cells with fisetin treatment (Fig. 2d). To figure out the mechanism of DNA damage induced by fisetin, we analyzed our previous RNA-sequencing results, GSE117189. The analysis identified 2672 differentially regulated genes (*P* < 0.05, *q* < 0.05, fold-change ≥ 2), of which 1136 and 1536 were up- and downregulated, respectively (Fig. 2e). We found that the mRNA levels of *RFXAP* and diverse DNA repair genes such as *RAD50*, *BRCA2*, *RPA2*, and *POLD3* were upregulated. We further confirmed the results of RNA sequencing by quantitative RT-PCR (qRT-PCR) (Fig. 2f).

### Expression of RFXAP in pancreatic cancer

**Fig. 3:**
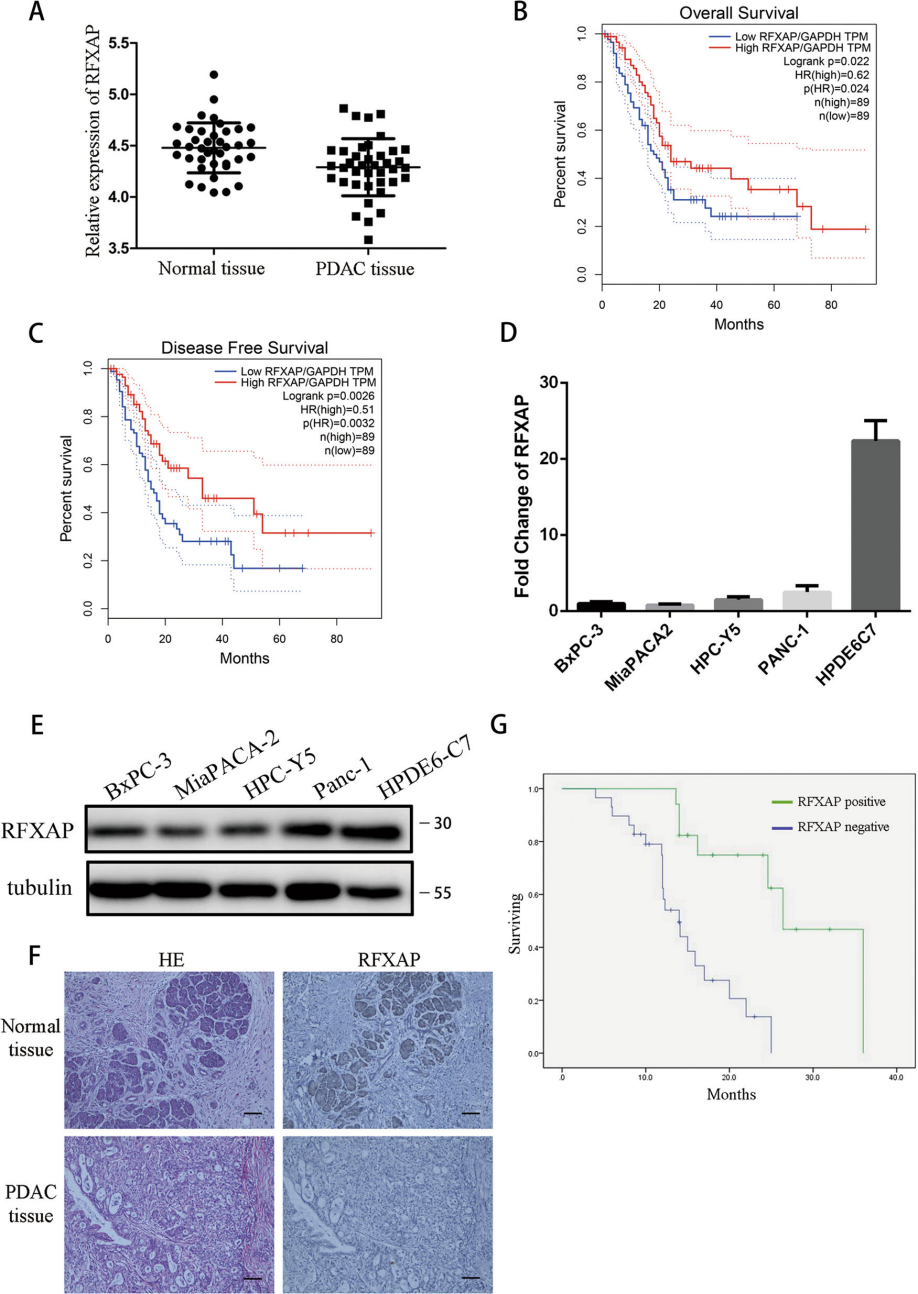
Expression of RFXAP in pancreatic cancer and its association with prognosis. **a** The Gene Expression Omnibus database (GSE15471) showed that the expression of RFXAP is lower in pancreatic cancer than in normal tissues. **b, c** The overall survival and disease-free survival analysis of patients with PDAC from GEPIA database. Patients with high levels of RFXAP have a better prognosis. Data are presented as mean ± SD (*n* = 89). **d** qRT-PCR showed that the mRNA expression of RFXAP was lower in pancreatic cancer-cell lines than in normal pancreatic ductal epithelial cells. Data are presented as mean ± SD (*n* = 3). **e** Western blot analysis showed that the expression of RFXAP was significantly lower in pancreatic cancer-cell lines than in normal pancreatic ductal epithelial cells. **f** The expression level of RFXAP in pancreatic cancer tissues and normal tissues was detected by immunohistochemistry. Scale bars 50 μm. **g** Expression of RFXAP in pancreatic cancer and its association with prognosis. Data are presented as mean ± SD.

A search of the Gene Expression Omnibus database (GSE15471) showed that the relative expression of *RFXAP* in PDAC was significantly lower than that in normal pancreatic tissues (4.29 vs. 4.48) (*P* < 0.05) (Fig. 3a). Based on Gene Expression Profiling Interactive Analysis (GEPIA)^28^, which is a newly developed interactive web server for analyzing the RNA-sequencing data of tumors and normal samples from The Cancer Genome Atlas (TCGA) and The Genotype-Tissue Expression databases based on a standard processing pipeline, we found that *RFXAP* expression was significantly reduced in high-grade (stage III and IV) tumors compared with low-grade (stage I and II) tumors, and the level of *RFXAP* was positively correlated with overall survival and disease-free survival (*P* < 0.05) (Fig. 3b, c and Supplementary Fig. S1C).

We used qRT-PCR to determine the mRNA levels of *RFXAP* in four pancreatic cancer-cell lines (BxPC-3, MiaPACA-2, HPC-Y5, and PANC-1) and one normal pancreatic ductal epithelial cell line (HPDE6-C7). *RFXAP* expression was significantly lower in the pancreatic cancer-cell lines than in HPDE6-C7 cells (Fig. 3d, e).

A total of 46 pancreatic cancer tissue specimens collected in our hospital were compared with 20 paracancerous tissue specimens as the control group. Immunohistochemistry was used to detect the expression of RFXAP in tissue samples, which showed that the RFXAP-positivity rate was significantly lower in pancreatic cancer samples than in the control group [37.0% (17/46) vs. 65.0% (13/20); *P* < 0.05] (Fig. 3f). Analysis of clinicopathological data showed that negative expression of RFXAP was significantly correlated with high TNM stage, T stage, and poor prognosis (Table 1 and Fig. 3g).

**Table 1:** Relationship between RFXAP expression and clinicopathological features.

Parameters	(*n*)	RFXAP expressions		*P* values (*<0.05)
		−	+	
Age				0.683
<60	18	12	6	
≥60	28	17	11	
Gender				0.936
Male	22	14	8	
Female	24	15	9	
TNM stage				0.012*
I–II	30	15	15	
III–IV	16	14	2	
Pathological grade				0.322
Poor	20	11	9	
Middle and high	26	18	8	
T stage				0.03*
T1, 2	13	5	8	
T3, 4	33	24	9	
Lymph node metastasis				0.322
No	26	18	8	
Yes	20	11	9	
Distant metastasis				0.366
M0	38	23	15	
M1	8	6	2	

### RFXAP functions in DNA damage induced by fisetin

**Fig. 4:**
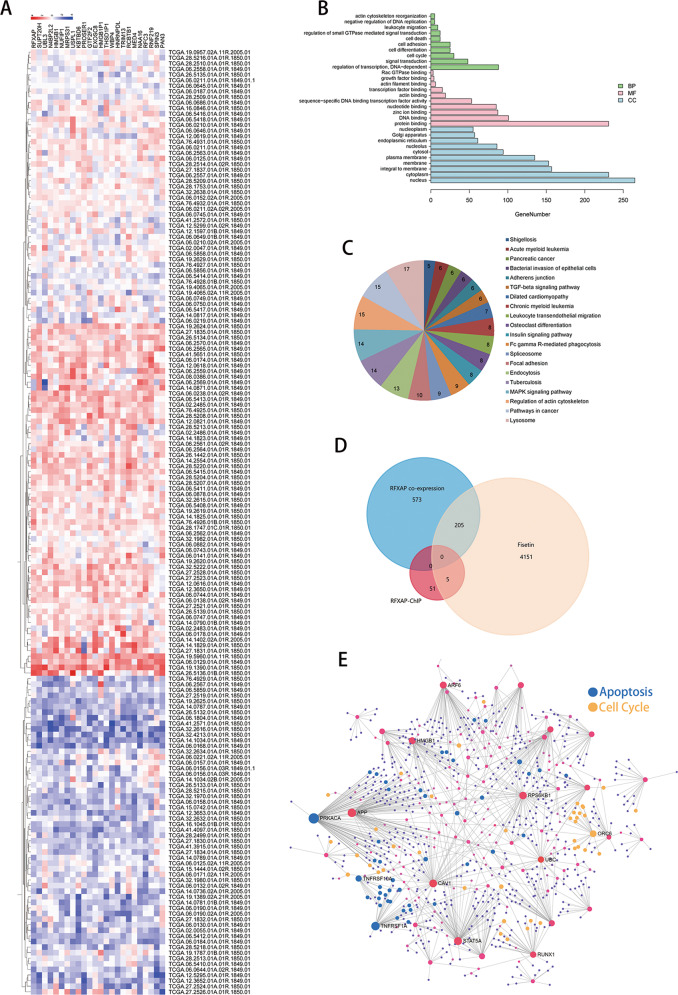
Analysis of genes coexpressed with RFXAP, ChIP sequencing, and RNA sequencing in pancreatic cancer. **a** Heat map of genes with a similarity coefficient of >0.5 with RFXAP in The Cancer Genome Atlas pancreatic cancer database. **b, c** RFXAP expression level was associated with cell-cycle regulation and DNA-damage response. **d** Overlapping genes of RFXAP co-expression, ChIP sequencing, and fisetin RNA sequencing were showed by Venn diagrams. **e** Protein–protein interaction of the overlapping genes of *RFXAP* co-expression and fisetin RNA-sequencing results was analyzed by NetworkAnalyst. Apoptosis and cell-cycle pathway were enriched, blue means genes related with apoptosis pathway, and yellow means genes related with cell-cycle pathway.

**Fig. 5:**
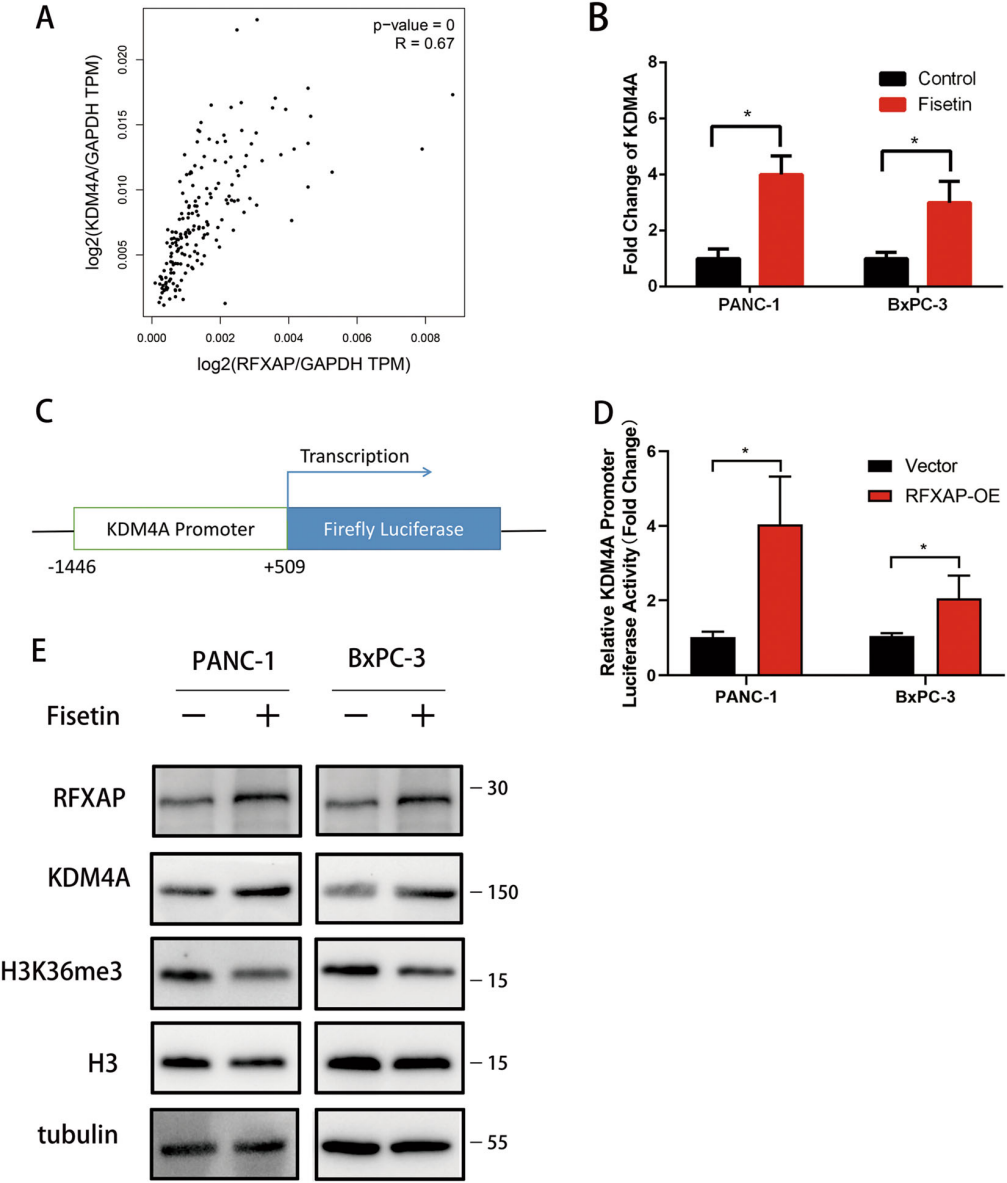
Expression of KDM4A was upregulated by fisetin. **a** RFXAP was positively correlated with the expression levels of KDM4A after normalized by GAPDH (*R* = 0.67, ****P < 0.0001, *n* = 89). **b** mRNA expressions of KDM4A in the pancreatic cancer-cell lines PANC-1 and BxPC-3 with treatment of fisetin were detected by qRT-PCR. Data are presented as mean ± SD (*n* = 3), *P < 0.05. **c, d** Transcriptional activity of *KDM4A* in RFXAP overexpressing and control PDAC cell lines. PANC-1 and BxPC-3 cells were transfected with control vector or RFXAP overexpression vector, then co-transfected with pGL4.23-KDM4A-promoter (−1446 to +509) and pRL-TK vectors. Luciferase activity was determined 24 h after transfection. Data are presented as mean ± SD (*n* = 3). **e** Expressions of RFXAP, KDM4A, and H3K36me3 in PDAC cells with fisetin treatment were detected by western blot analysis.

To investigate the molecular mechanisms underlying the role of RFXAP in PDAC, genes coexpressed with *RFXAP* were identified in TCGA pancreatic cancer database. Twenty-three genes were significantly coexpressed with *RFXAP* (*R* > 0.5), and 778 genes were coexpressed with an *R* value > 0.3 (Supplementary Table 1). The heat map for coexpressed genes is shown in Fig. 4a. Gene ontology and KEGG were used to predict signaling pathways associated with *RFXAP* (Fig. 4b, c and Supplementary Tables 2 and 3), and showed that *RFXAP* expression was correlated with cell-cycle regulation and the DNA-damage response. *RFXAP* was positively correlated with *RAD50*, *BRCA2*, *RPA2*, *POLD3*, and other genes (Supplementary Fig. S1D). These genes play important roles in DNA-damage repair, whereas dysregulation of their expression often results in pancreatic cancer gene mutations, chemoradiotherapy resistance, and tumor progression^29–41^. To further examine the role of *RFXAP* in PDAC cells with treatment of fisetin, ChIP sequencing was performed in RFXAP-overexpressing PANC-1 (RFXAP-PANC-1) cells to explore the function of RFXAP as a transcription factor. The results showed that RFXAP interacted with the promoters of 65 genes (Table 2 and Supplementary Table 4). We further analyzed the RNA-sequencing results and found that there were 4361 differentially regulated genes (*P* < 0.05, *q* < 0.05, fold-change > 1.5). Then we analyzed the overlapping genes of *RFXAP* co-expression, ChIP sequencing, and fisetin RNA sequencing by Venn diagrams (Fig. 4d). Based on NetworkAnalyst (http://www.networkanalyst.ca/)^42^, we analyzed the protein–protein interaction of the overlapping genes of *RFXAP* co-expression and fisetin RNA-sequencing results and found that apoptosis and cell-cycle pathways were enriched (Fig. 4e and Supplementary Table 5). We focused on *KDM4A* for the analysis of overlapping genes of ChIP sequencing and RNA sequencing. Recent studies showed that KDM4A can impair DNA HR repair through regulation of histone H3K36 demethylation^10^. Based on the GEPIA analysis, we found that *RFXAP* was positively correlated with *KDM4A* after normalization with *GAPDH*, and patients with high *KDM4A* expression showed better prognoses (Fig. 5a and Supplementary Fig. S1E, F). Thus, we hypothesized that RFXAP might be involved in fisetin-induced DNA damage in PDAC. qRT-PCR analysis further verified that *KDM4A* was upregulated in PDAC treated with fisetin (Fig. 5b). Dual-luciferase reporter assays further identified the transcriptional regulation of *KDM4A* by RFXAP in both PANC-1 and BxPC-3 cells (Fig. 5c, d). We also detected the protein level of KDM4A and histone H3K36me3, which can be attenuated by KDM4A, in PDAC cells with fisetin treatment. The results showed that fisetin reduced the expression of H3K36me3 (Fig. 5e).

**Table. 2:** RFXAP interacted with the promoters of 65 genes.

RefSeq ID	Symbol	RefSeq ID	Symbol
NM_001190452	MTRNR2L1	NR_024231	SNAR-B1
NM_001190470	MTRNR2L2	NR_024242	SNAR-A14
NM_001190702	MTRNR2L8	NR_026999	LINC00265
NM_001286136	ODF3	NR_027020	ANKRD30BL
NM_001293091	CDC27	NR_027412	LINC00910
NM_004533	MYBPC2	NR_029889	MIR337
NM_014663	KDM4A	NR_030386	MIR663A
NM_017761	PNRC2	NR_030617	MIR665
NM_018093	WDR74	NR_031608	MIR663B
NM_152837	SNX16	NR_033770	ROCK1P1
NR_003594	REXO1L2P	NR_037421	MIR3648-1
NR_024214	SNAR-A3	NR_037458	MIR3687-1
NR_024215	SNAR-A4	NR_038368	LINC00273
NR_024221	SNAR-C3	NR_039666	MIR4461
NR_024223	SNAR-A5	NR_039689	MIR4477B
NR_024224	SNAR-A7	NR_039705	MIR4485
NR_024225	SNAR-A11	NR_040095	MIR663AHG
NR_024226	SNAR-A9	NR_046018	DDX11L1
NR_024227	SNAR-A6	NR_047479	LINC00854
NR_024228	SNAR-A8	NR_051985	DDX11L9
NR_024229	SNAR-A10	NR_103714	FAM27E2
NR_024230	SNAR-B2	NR_103772	LOC100288162
NR_106735	MIR6087	NM_001114091	CDC27
NR_106781	MIR6723	NM_001256	CDC27
NR_107045	MIR8078	NM_001293089	CDC27
NR_108107	FAM230B	NM_001307977	WDR74
NR_110795	LOC102723376	NM_001308208	ARHGAP39
NR_121645	RNVU1-8	NM_022133	SNX16
NR_121647	LINC01410	NM_053280	ODF3
NR_125957	LOC101928626	NM_152836	ODF3
NR_128711	MIR3648-2	NR_027413	LINC00910
NR_128714	MIR3687-2	NR_034090	DDX11L9
NR_132380	LINC01596

### RFXAP regulates DNA damage via KDM4A-dependent H3K36 demethylation

**Fig. 6:**
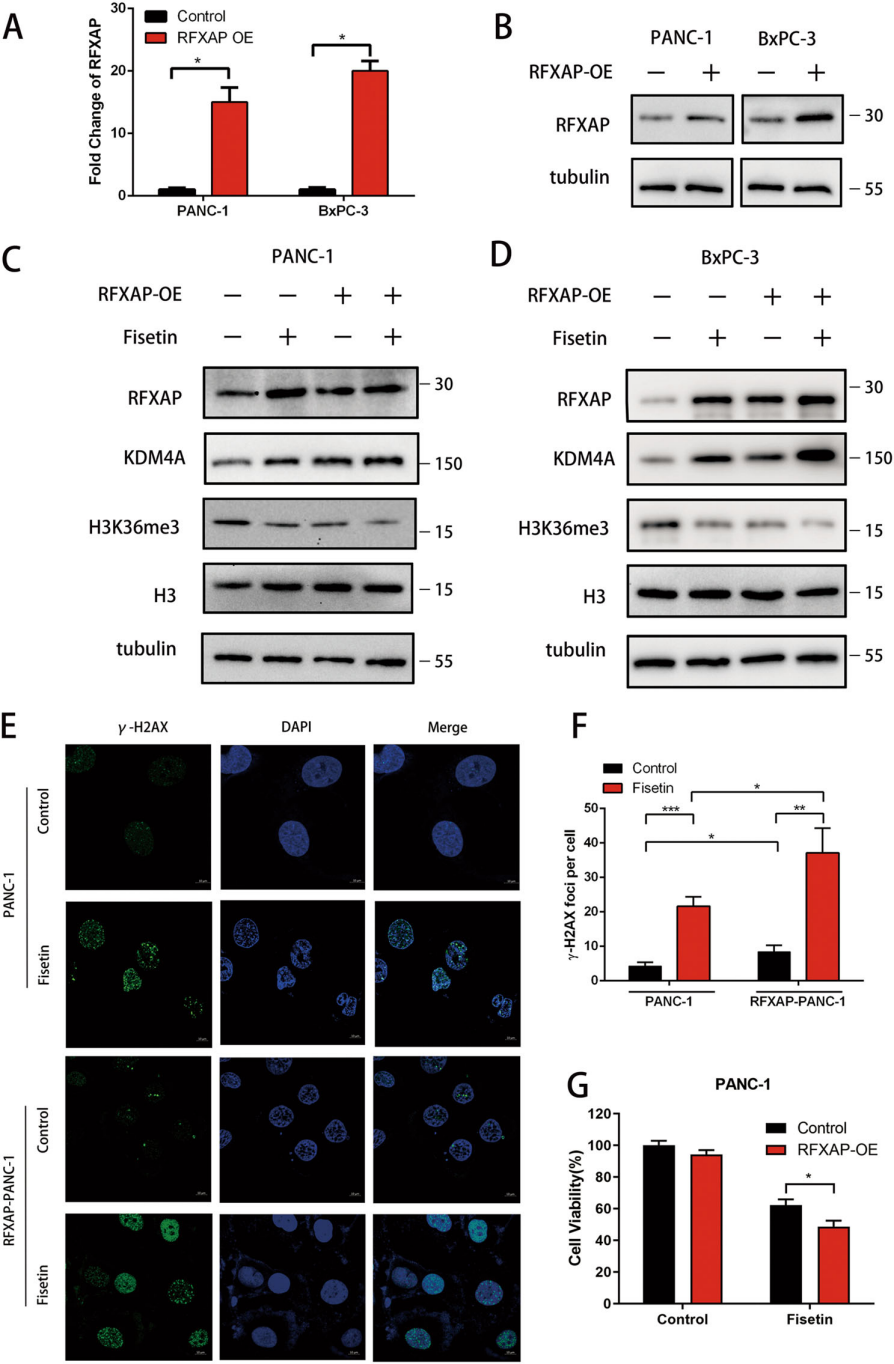
RFXAP overexpressing enhanced the DNA damage induced by fisetin in pancreatic cancer. **a** qRT-PCR of RFXAP in control and RFXAP overexpression (RFXAP-OE) PANC-1 and BxPC-3 cells with 100, 50 μM fisetin treatment for 48 h. respectively. The results are presented as the mean ± SD (*n* = 3), *P < 0.05. **b** RFXAP protein expressions were detected by western blot analysis. **c, d** Expressions of RFXAP, KDM4A, and H3K36me3 in control and RFXAP overexpression PDAC cells with or without fisetin treatment. **e** Analyses of DNA damage by observation of the immunofluorescence of γ-H2AX (green) in PDAC cells. RFXAP-PANC-1 cells were treated with fisetin (100 μM) for 48 h. The nucleus were counterstained with DAPI (blue). Scale bars 10 μm. **f** Comparative changes in the γ-H2AX spots number in control and RFXAP-PANC-1 cells with treatment groups. The spots were counted by manual scoring. Results are mean ± SD, *P < 0.05, **P < 0.01, ***P < 0.001. **g** CCK-8 asssy of RFXAP-overexpressing PANC-1 cells. Cells were treated with fisetin (0 or 100 μM) for 48 h, respectively. The absorbance was measured at 450 nm. Data are presented as mean ± SD (*n* = 3); *P < 0.05.

**Fig. 7:**
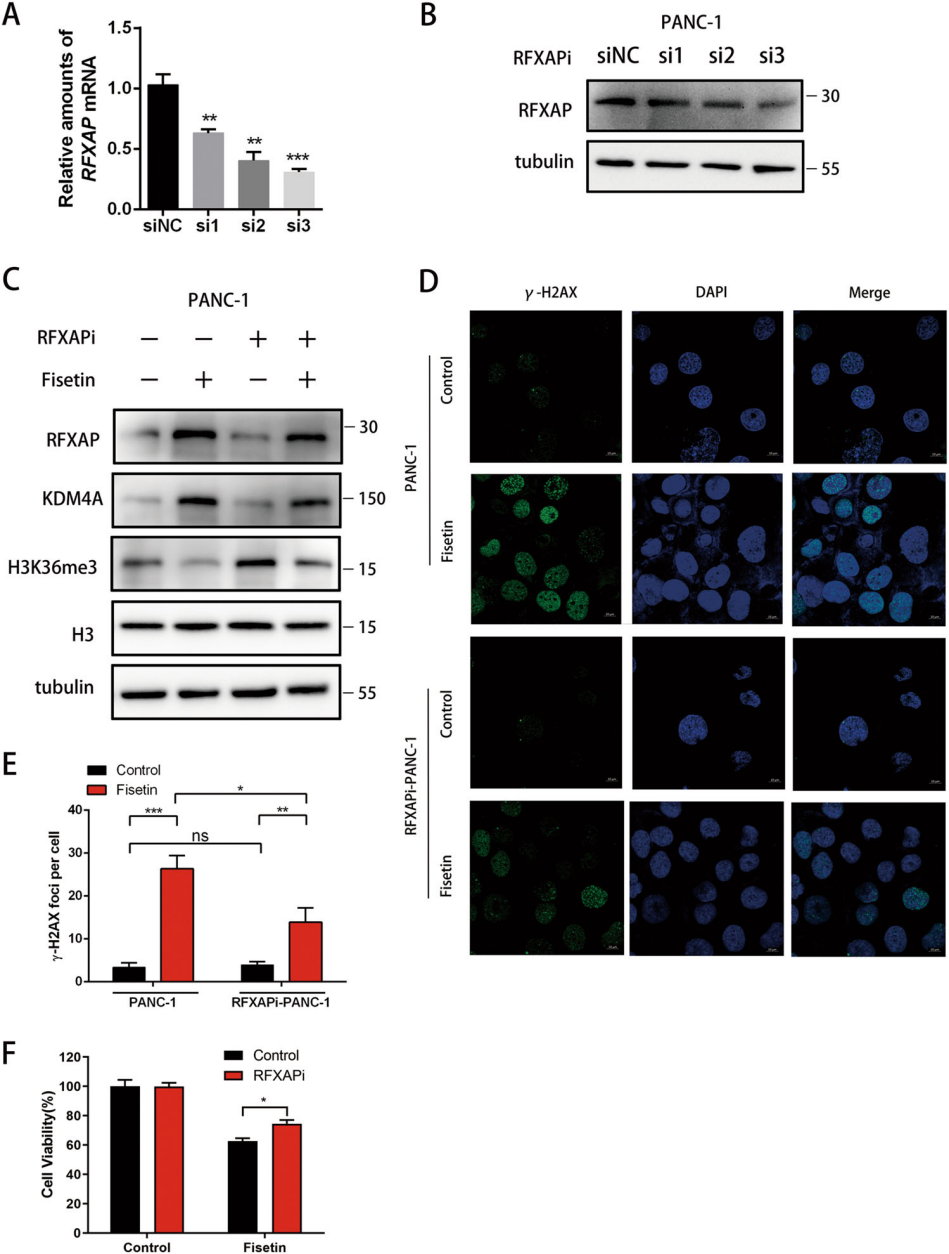
RFXAP silencing attenuated the DNA damage induced by fisetin in pancreatic cancer. **a** mRNA levels of RFXAP in control and RFXAP silencing (si1, si2, si3) PANC-1 were detected by qRT-PCR. The results are presented as the mean ± SD (*n* = 3), **P < 0.01. ***P < 0.001. **b** RFXAP protein expressions in RFXAP silencing PANC-1 cells were detected by western blot analysis. **c** Expression levels of KDM4A and H3K36me3 in RFXAP silencing PANC-1 cells, with or without fisetin treatment, were detected by western blot analysis. **d** Analyses of DNA damage by observation of the immunofluorescence of γ-H2AX (green) in PDAC cells. RFXAPi-PANC-1 cells were treated with fisetin (100 μM) for 48 h. The nucleus were counterstained with DAPI (blue). Scale bars 10 μm. **e** Comparative changes in the γ-H2AX spots number in control and RFXAPi-PANC-1 cells with treatment groups. The spots were counted by manual scoring. Results are mean ± SD, *P < 0.05, **P < 0.01, ***P < 0.001, #ns no significance. **f** CCK-8 asssy of RFXAP silencing PANC-1 cells. Cells were treated with fisetin (0 or 100 μM) for 48 h, respectively. The absorbance was measured at 450 nm. Data are presented as mean ± SD (*n* = 3); *P < 0.05.

To further confirm whether the DNA damage observed following fisetin treatment was associated with RFXAP, we overexpressed *RFXAP* by pcDNA-RFXAP plasmids. qRT-PCR and western blot analysis showed the efficiency of *RFXAP* overexpression (Fig. 6a, b). Then we found that *RFXAP* overexpression enhanced the levels of KDM4A and attenuated the expression of H3K36me3 (Fig. 6c, d). Moreover, immunofluorescence showed that overexpression of RFXAP enhanced the gamma-H2AX foci levels after fisetin treatment (Fig. 6e, f), and CCK-8 assay showed that overexpression of RFXAP strengthened the antitumor effect of fisetin (Fig. 6g). Then we silenced *RFXAP* expression by RNAi; RFXAP expression was detected by qRT-PCR and western blot analysis. The results revealed that expression of *RFXAP* was reduced by silencing (Fig. 7a, b). Then we picked most-efficient RFXAP silencing siRNA (si3) to further test the effects of RFXAP silencing by western blot analysis. The results indicated that *RFXAP* silencing attenuated the expression of KDM4A and enhanced the levels of H3K36me3 (Fig. 7c). Immunofluorescence revealed that *RFXAP* silencing reduced gamma-H2AX foci levels (Fig. 7d, e), and RFXAP silencing attenuated the growth inhibition effect of fisetin in PDAC cells (Fig. 7f). To further determine the role of KDM4A in pancreatic cancer cells, we depleted *KDM4A* with CRISPR/Cas9 in PANC-1 cells. Western blot analysis showed the depletion of *KDM4A* and upregulation of H3K36me3 in PANC-1 cells with or without fisetin treatment (Supplementary Fig. S2A). We also found that depletion of KDM4A enhanced cell viability of PANC-1 cells with fisetin treatment (Supplementary Fig. S2B). Immunofluorescence revealed that depletion of *KDM4A* enhanced H3K36me3 levels and reduced gamma-H2AX foci levels (Supplementary Fig. S2C, D). Together, these results showed that *RFXAP* enhanced the DNA damage induced by fisetin via regulation of KDM4A and H3K36me3, thereby impairing DNA HR repair.

**Fig. 8:**
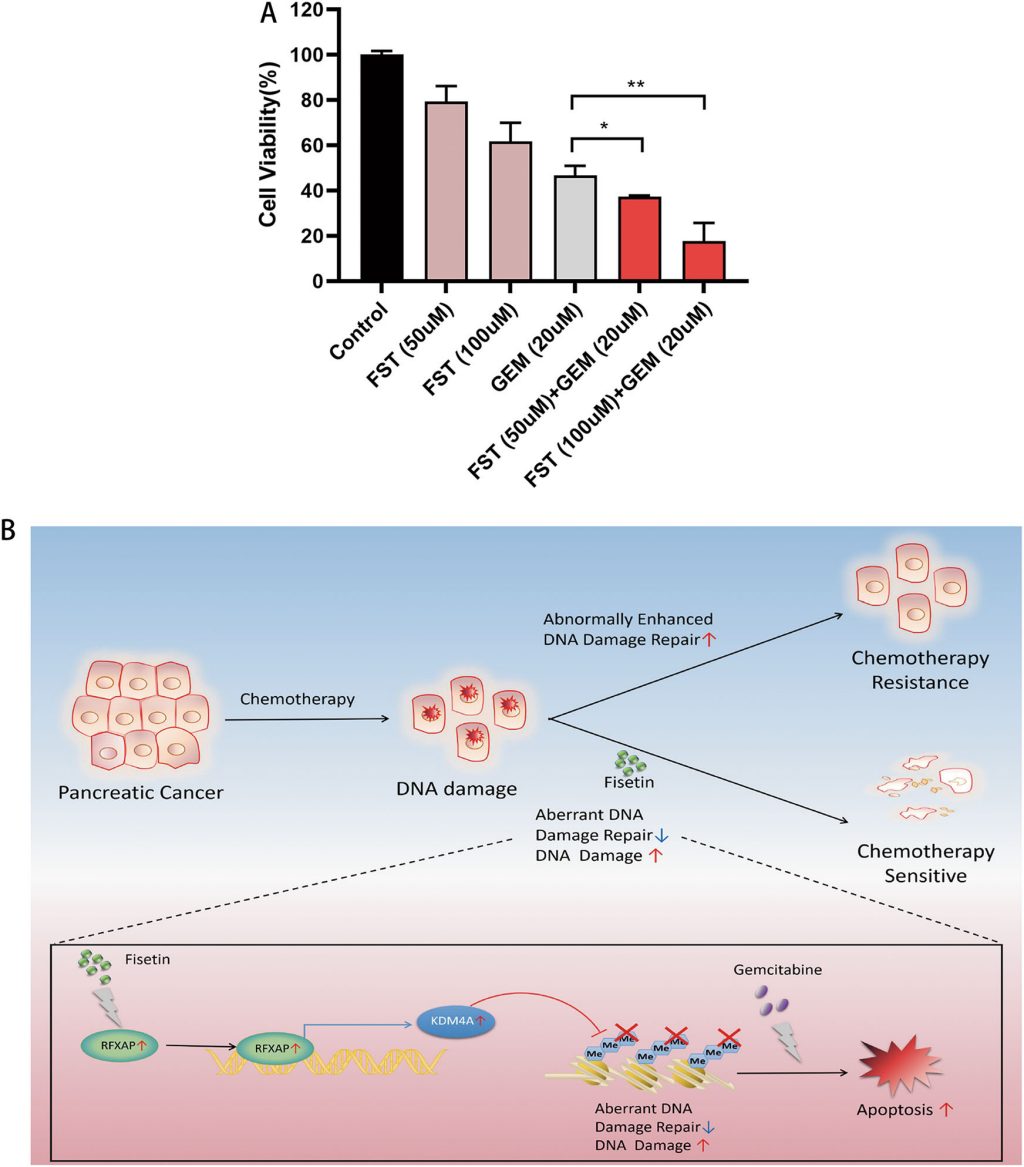
Fisetin enhances chemosensitivity in pancreatic cancer. **a** PANC-1 cell viability decreased significantly after co-treatment with 20-μM gemcitabine and fisetin (50 or 100 μM). Data are presented as the mean ± SD (*n* = 3). **b** Model indicating that the regulation of DNA damage via RFXAP/KDM4A-dependent histone H3K36 demethylation signaling by fisetin leads to drug sensitivity in pancreatic cancer.

Moreover, we found the function of fisetin in enhancing the effect of chemotherapy on pancreatic cancer cells. The results showed that PANC-1 cell viability decreased significantly after co-treatment with 20-μM gemcitabine and fisetin (50 or 100 μM) (Fig. 8a), which showed that fisetin might have a potential to be used as a complementary therapy in cases of chemotherapy resistance by enhancing the chemosensitivity of pancreatic cancer cells.

## Discussion

The low rate of early diagnosis and radical resection of pancreatic cancer remains one of the major challenges faced by clinical oncologists and surgeons^1^. More than 80% of PDAC patients are at an advanced stage and considered unresectable at the time of diagnosis. Although gemcitabine, S-1, albumin-bound paclitaxel, and other chemotherapy drugs can, to some extent, prolong the survival time of patients with advanced PDAC, after radio-chemotherapy, the median survival time of the unresectable patients generally does not exceed 1 year. The efficacies of radiotherapy, targeted therapy, and immunotherapy are also unsatisfactory^43–46^. The nucleotide analogue gemcitabine, which causes cytotoxicity by inducing DNA damage, S-phase arrest, and apoptosis of cancer cells, remains the standard chemotherapy for PDAC^47^. However, acquired drug resistance to gemcitabine is a major obstacle of chemotherapy for pancreatic cancer^48^. Drug resistance of gemcitabine might be associated with the elevated DNA repair capacity of PDAC^6,7,48^. Thus, effective targeted therapy or other novel therapies are urgently needed for the treatment of pancreatic cancer.

RFXAP can regulate the transcription of MHC II molecules by binding to CIITA, RFXANK, and RFX5 to form RFX complexes^49,50^. We previously found that, in PDAC, miR-212-3p could be transferred into dendritic cells via exosomes to inhibit RFXAP and MHC class II molecules in dendritic cells, thereby inducing immune tolerance^10^. In the current study, *RFXAP* expression was significantly lower in pancreatic cancer-cell lines and tumor tissues than in the corresponding controls, and negative *RFXAP* expression was significantly correlated with TNM stage, T stage, and prognosis. These findings suggest that RFXAP deficiency is a characteristic of PDAC and the expression of RFXAP might be a potential marker of prognosis of PDAC patients.

Fisetin is a natural flavonoid that has been widely studied and inhibits the growth and proliferation of diverse cancer-cell lines. The antitumor effects of fisetin have been indicated to be mediated by altering different signaling pathways in different cellular contexts. Recent studies revealed that fisetin can induce DNA damage and apoptosis in gastric and hepatic cancer^25,26^. Here we found that fisetin induced DNA damage and S-phase arrest, which was similar to the effect of gemcitabine. However, the fisetin molecule differs to the nucleotide analogue gemcitabine. Thus, we hypothesized that fisetin might induce DNA damage through different mechanisms. RNA sequencing found that *RFXAP* and some genes involved in DNA-damage response, such as *RAD51*, *RAD50*, *BRCA2*, *RPA2*, and *POLD3* were upregulated by fisetin. Bioinformatics analysis showed a correlation between RFXAP and DNA repair genes (i.e., RFXAP expression is positively correlated with *RAD50*, *BRCA2*, *RPA2*, and *POLD3* expression)^29–41^. We further explored the role of RFXAP as a transcription factor using ChIP sequencing. We analyzed overlapping genes of RFXAP co-expression, ChIP sequencing, and fisetin RNA sequencing, and found that RFXAP targeted *KDM4A*, a specific demethylase for tri- and dimethylated histone H3K36 and H3K9, which can impair DNA HR repair via regulation of H3K36 methylation^10,51^. H3K36me3 is required for HR repair due to its recruitment effect on lens epithelium-derived growth factor p75 (LEDGF). Following DNA DSBs, LEDGF recruits CtIP to promote resection, and then recruits RPA and RAD51 during HR repair^10,11,52,53^. KDM4A overexpression reduces global H3K36me3 levels, and thereby inhibits RAD51 and RPA foci following DNA damage, which indicates the impairment of HR repair^10^. Then we found that KDM4A was upregulated by fisetin in PDAC, and RFXAP overexpression could enhance the DNA damage via upregulated KDM4A, thereby attenuating the level of H3K36me3. Moreover, depletion of *KDM4A* showed lower gamma-H2AX foci levels together with higher H3K36me3 levels and cell viability in PDAC cells with fisetin treatment. We also revealed that *RFXAP* silencing could downregulate the DNA damage induced by fisetin, via KDM4A-dependent regulation of H3K36 methylation. PDAC is an extremely malignant tumor with genetic instability, which leads to high levels of DNA damage, elevated DNA-damage repair, and serious drug resistance. The deficiency of RFXAP might play an important role in DNA repair and drug resistance. And our results also showed that fisetin might have a potential to be used as a complementary therapy by enhancing the chemosensitivity of pancreatic cancer cells.

In summary, the expression of RFXAP, which was deficient in PDAC, was positively correlated with survival of PDAC patients. Fisetin increased the expression of RFXAP and KDM4A, which impaired HR repair via regulation of histone H3K36 methylation and might attenuate the excessive DNA repair of PDAC, thereby inducing DNA damage and S-phase arrest of PDAC. Thereby fisetin enhances the drug sensitivity of gemcitabine in pancreatic cancer. Together these results indicate that fisetin inhibits cancer-cell proliferation by inducing DNA damage via RFXAP/KDM4A-dependent demethylation of histone H3K36, which might be a novel therapeutic target for PDAC.

## Supplementary information

Figure.S1

Figure.S2

Supplementary figure legend

Supplement Table 1

Supplement Table 2

Supplement Table 3

Supplement Table 4

Supplement Table 5
