# Supplementary figures and images for: Fisetin inhibits proliferation of pancreatic adenocarcinoma by inducing DNA damage via RFXAP/KDM4A-dependent histone H3K36 demethylation
Source: Cell Death Dis. 2020 Oct 22;11(10):893. doi: 10.1038/s41419-020-03019-2 (PMC7582166; doi:10.1038/s41419-020-03019-2)

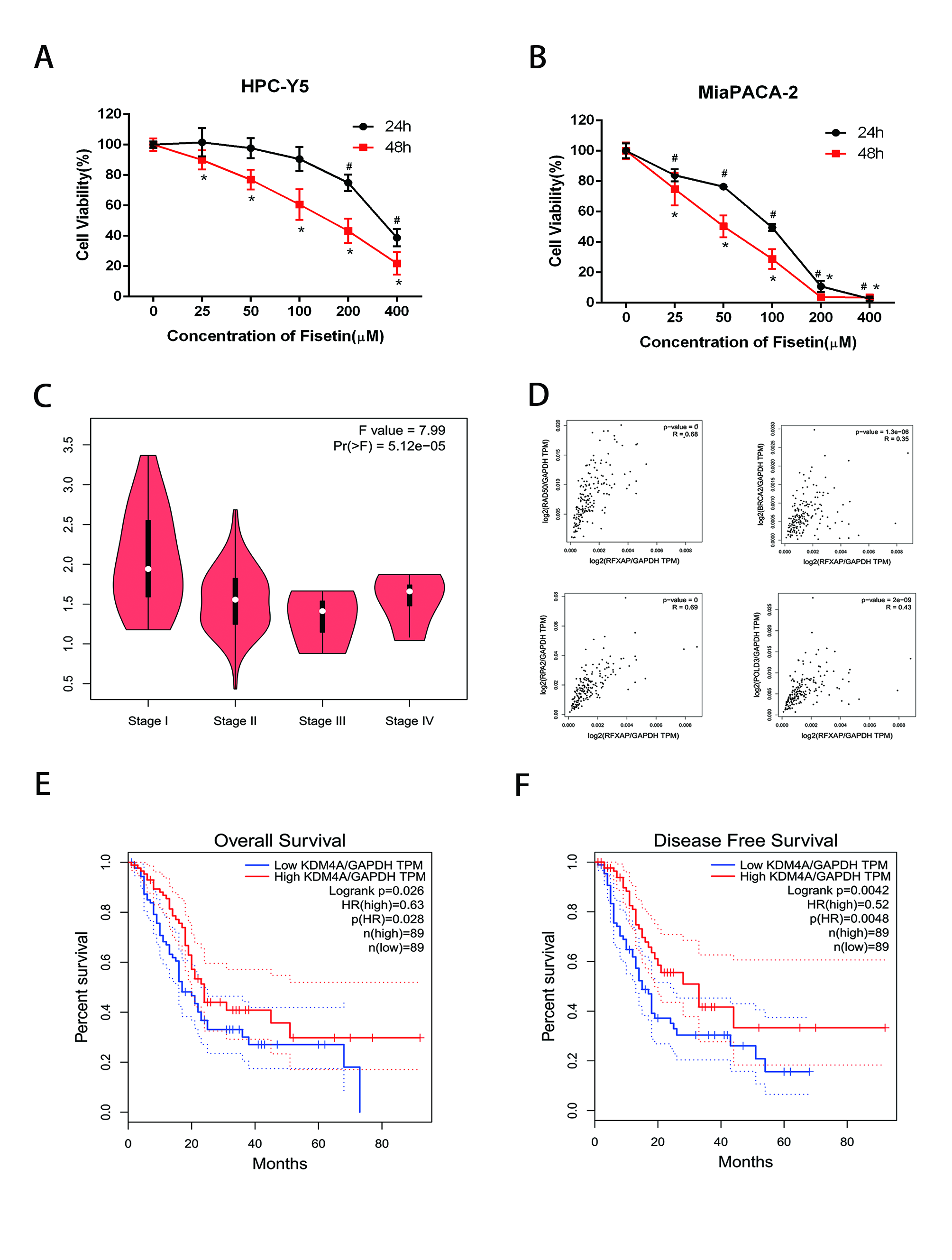

Supplement: Supplementary file 1 — Figure.S1 [file 41419_2020_3019_MOESM1_ESM.tif]

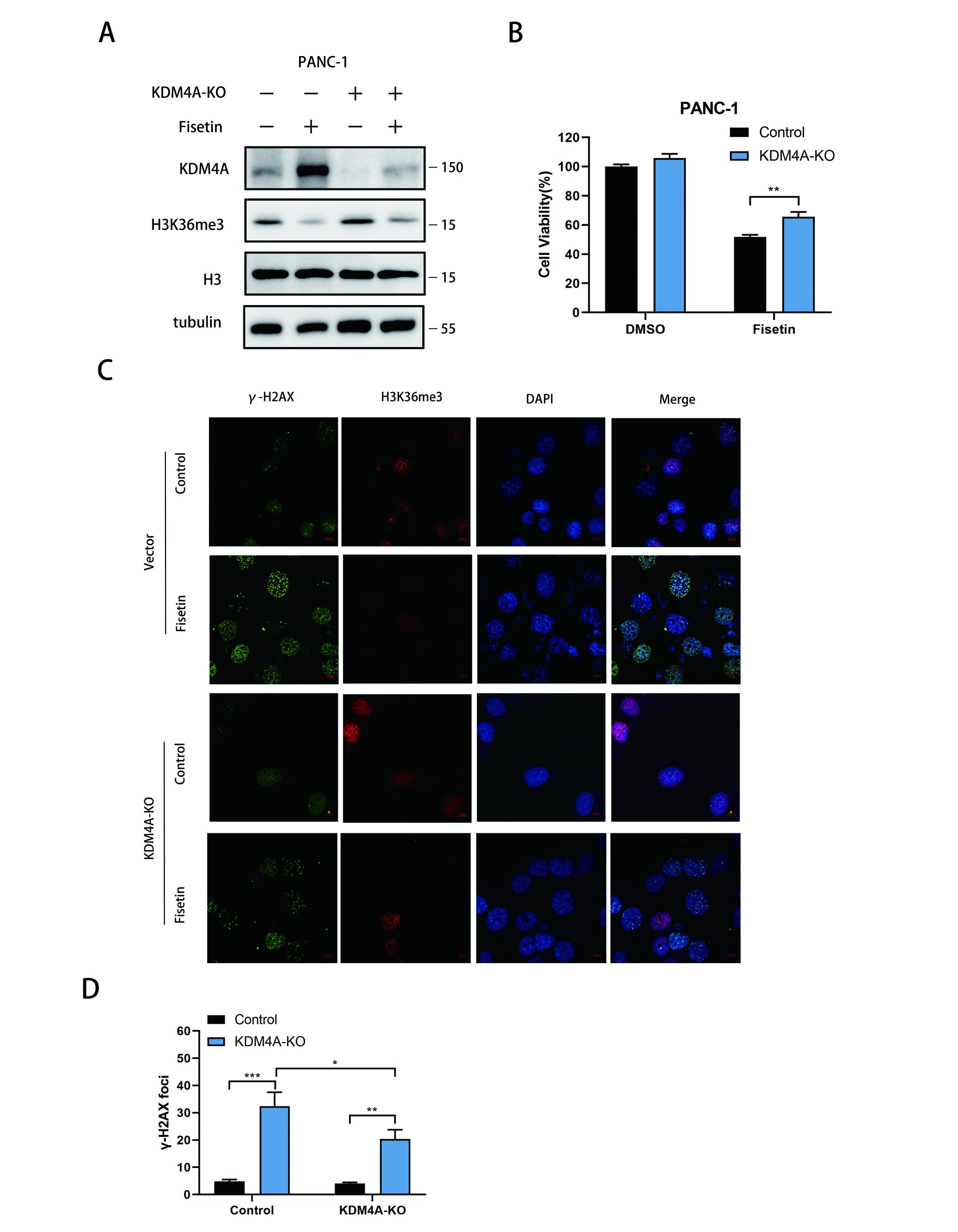

Supplement: Supplementary file 2 — Figure.S2 [file 41419_2020_3019_MOESM2_ESM.tif]
