# Supplementary material for: Fisetin inhibits proliferation of pancreatic adenocarcinoma by inducing DNA damage via RFXAP/KDM4A-dependent histone H3K36 demethylation
Source: Cell Death Dis. 2020 Oct 22;11(10):893. doi: 10.1038/s41419-020-03019-2 (PMC7582166; doi:10.1038/s41419-020-03019-2)
Supplement: Supplementary file 3 — Supplementary figure legend [file 41419_2020_3019_MOESM3_ESM.doc]

**Fig. S1.** **a,b** CCK-8 asssy of HPC-Y5 and MiaPACA-2 cells. Cells were treated with fisetin (0-400μM) for 24 h, 48 h, respectively. The absorbance was measured at 450nm. Data are presented as mean ± SD (n=3); #P<0.05 for group 24 h, *P<0.05 for group 48 h. **c** RFXAP expression stage plot of PDAC patients based on GEPIA analysis. **d** RFXAP was positively correlated with the expression levels of RAD50, BRCA2, RPA2, POLD3. **e,f** The overall survival and disease free survival analysis of patients with PDAC from GEPIA database. Patients with high levels of KDM4A have a better prognosis. Data are presented as mean ± SD (n=89).

**Fig. S2.** **a** Expressions of KDM4A and H3K36me3 in control and KDM4A-knockout (KDM4A-KO) PDAC cells with or without fisetin treatment. **b** CCK-8 asssy of *KDM4A*-knockout PANC-1 cells. Cells were treated with fisetin (100μM) for 48 h. The absorbance was measured at 450nm. Data are presented as mean ± SD (n=3); **P<0.01. **c** Analyses of DNA damage by observation of the immunofluorescence of γ-H2AX(green) and H3K36me3(red) in PDAC cells. Cells were treated with fisetin (100 μM) for 48h. The nucleus were counter stained with DAPI (blue). Scale bars 10μm. **d** Comparative changes in the γ-H2AX spots number in control and KDM4A-KO PANC-1 cells with treatment groups. The spots were counted by manual scoring. Results are mean ± SD(n=5), *P<0.05, **P<0.01, ***P<0.001.
